# The Connection of Immune Response and cGAS-STING Pathway in Cardiovascular Disease: From Basic Mechanism to Therapeutic Implications

**DOI:** 10.3390/biomedicines14102278

**Published:** 2026-10-08

**Authors:** Xiaoyan Wang, Yang Xu, Chongzhe Pei, Hongmei Zheng, Heng Cai

**Affiliations:** 1 Institutes of Biomedical Sciences, Fudan University, No. 131 Dong’an Road, Xuhui District, Shanghai 200032, China; 2 Department of Cardiology, Zhongshan Hospital, Fudan University, No. 180 Fenglin Road, Xuhui District, Shanghai 200032, China; 3 Department of Cardiology, Tianjin Medical University General Hospital, No. 154 Anshan Road, Heping District, Tianjin 300052, China; yuong1101@163.com (Y.X.);

**Keywords:** immune response, DNA damage, cGAS-STING pathway, cardiovascular disease, precision medicine, innate immunity

## Abstract

The escalating global burden of cardiovascular disease (CVD) necessitates a deeper elucidation of the fundamental molecular mechanisms driving its pathogenesis. Aberrant immune activation, specifically sterile inflammation, is now recognized as a core hallmark of CVD, predominantly ignited by the pathological accumulation of damaged self-DNA. As the principal cytosolic DNA sensor, the cyclic GMP-AMP synthase (cGAS)–stimulator of interferon genes (STING) pathway has emerged as a crucial mechanistic nexus translating genomic distress into robust innate immune responses. Despite its paramount importance, a comprehensive integration of how DNA damage-induced cGAS-STING activation orchestrates cardiovascular immunology remains underexplored. In this review, we comprehensively synthesize the dynamic immunological landscape of CVD, with a specific focus on the critical interplay between DNA damage and sterile inflammation. We delineate the intricate molecular cascades of the cGAS-STING pathway across diverse cardiovascular pathologies and critically evaluate its role as a master amplifier of disease progression. Furthermore, we highlight the translational trajectory of targeting the cGAS-STING axis, emphasizing how deciphering this DNA damage-immune network provides a rational blueprint for developing precision immunomodulatory therapies in cardiovascular medicine.

## 1. Introduction

Despite modern therapeutic advances, cardiovascular disease (CVD) remains the leading cause of global mortality, currently accounting for over 20 million deaths annually worldwide and demonstrating a continuously ascending trajectory driven by aging populations and escalating metabolic risk factors [1]. Beyond traditional hemodynamic and metabolic risk factors, emerging paradigms identify the immune system and localized inflammatory cascades as fundamental drivers of cardiovascular pathogenesis [2,3]. This immunological involvement pervades the entire CVD spectrum, orchestrating disease progression in both acute infectious etiologies (e.g., myocarditis [4] and endocarditis [5]) and sterile conditions, ranging from myocardial infarction [6] and autoimmune cardiac diseases [7] to subsequent pathological ventricular remodeling [8,9].

Although clinical evidence underscores the therapeutic promise of targeting inflammation in CVD [10], the precise upstream triggers initiating these detrimental immune responses remain incompletely defined. In sterile cardiovascular injury, physiological stressors—such as ischemia, mechanical overload, and oxidative stress—provoke severe genomic and mitochondrial damage. The consequent breach of subcellular compartmentalization extrudes self-DNA into the cytosol, where it acts as a potent damage-associated molecular pattern (DAMP) that fundamentally activates innate immune cascades [11,12].

At the nexus of this DNA damage and immune activation lies the cyclic GMP-AMP synthase (cGAS)–stimulator of interferon genes (STING) pathway. Originally characterized as a primary defense against microbial pathogens, the cGAS-STING axis is now recognized as the master cytosolic sensor for mislocalized endogenous DNA [13]. By translating structural organelle distress into robust type I interferon responses and pro-inflammatory cytokine storms, this pathway drives chronic sterile inflammation and maladaptive cardiovascular remodeling [14,15,16].

In this review, we synthesize the evolving landscape of cardiovascular immunology, emphasizing the critical interplay between DNA damage and innate immune activation. We comprehensively delineate the molecular mechanisms governing the cGAS-STING axis across diverse cardiovascular pathologies and critically evaluate the translational potential of targeting this pathway. By bridging mechanistic insights with emerging pharmacological interventions, we highlight future avenues for precision immunomodulatory therapies in cardiovascular medicine.

## 2. The Inflammatory and Immunological Landscape of Cardiovascular Disease

**Figure 1:**
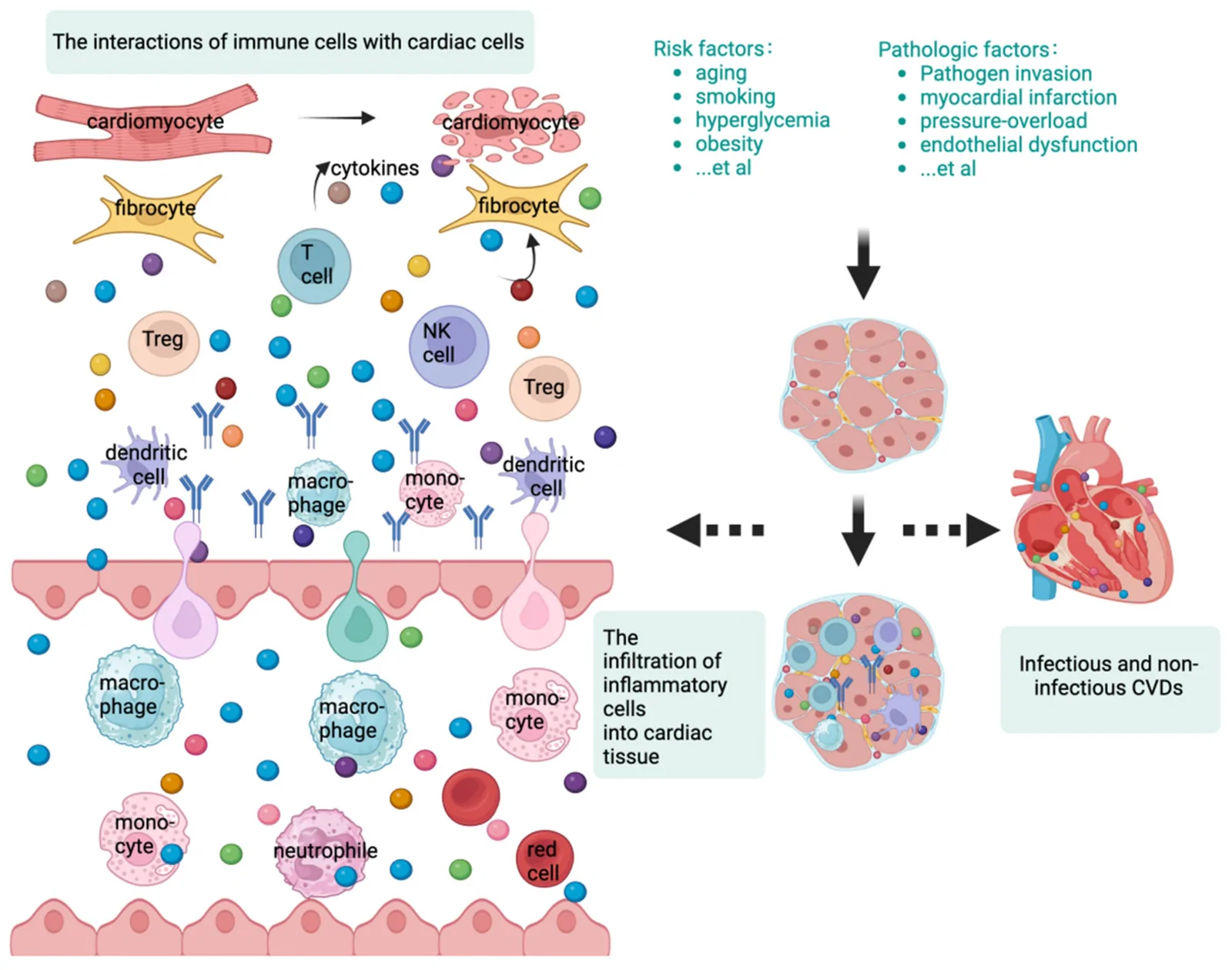
The immune response in cardiovascular disease. Both risk factors and pathologic factors could trigger inflammation. The inflammatory cells infiltrate into cardiac tissue and interact with cardiac cells, and those immune responses matter in both infectious and non-infectious CVDs. Black arrows indicate inflammation progression and immune cell infiltration; dashed arrows indicate the link between infiltration and CVDs; colored dots represent cytokines. Abbreviations: Treg, regulatory T cells; NK, natural killer.

Substantial clinical and experimental evidence [17,18] establishes inflammation as a fundamental driver of cardiovascular disease (CVD) pathogenesis, this immunological burden is bimodally distributed. Systemically, established risk factors—such as aging, smoking, hyperglycemia, and obesity—orchestrate a chronic, low-grade inflammatory milieu [19,20,21,22]. In this context, obesity-driven adipose tissue expansion recruits pro-inflammatory macrophages that amplify circulating cytokines, ultimately provoking endothelial dysfunction and accelerating atherogenesis [22]. Locally, acute pathological insults—ranging from infectious etiologies like viral myocarditis to sterile triggers such as pressure overload and myocardial infarction (MI)—precipitate intense localized inflammatory cascades (Figure 1). Following ischemic injury, necrotic cardiomyocytes rapidly extrude danger-associated molecular patterns (DAMPs), catalyzing robust leukocyte infiltration that governs adverse tissue remodeling [23,24].

The precise initiation and temporal trajectory of these immune responses dictate the clinical evolution of both infectious and sterile CVD [9,25]. In infectious myocarditis, pathogen invasion prompts the rapid recruitment of circulating leukocytes; while essential for microbial clearance, this robust macrophage and T-cell infiltration simultaneously drives off-target myocardial necrosis and maladaptive fibrotic remodeling [26,27,28]. Conversely, in sterile ischemic injury, DAMPs—particularly fragmented nucleic acids and mitochondrial constituents—released from necrotic cells rapidly mobilize circulating monocytes and neutrophils to the infarct core. This inflammatory infiltrate secretes cytokines, chemokines, and reactive oxygen species (ROS), which exacerbate the primary ischemic insult via reperfusion injury, progressive cardiomyocyte apoptosis, and extracellular matrix (ECM) degradation [29,30]. Furthermore, ischemia-recruited monocytes undergo epigenetic reprogramming—a phenomenon termed “trained immunity”—that not only perpetuates local tissue destruction but also sustains a systemic hyperinflammatory state, accelerating concurrent atherosclerosis [31]. Crucially, emerging literature [32] establishes a direct mechanistic link connecting this trained immunity to the cGAS-STING cascade. Following initial ischemic injury, the massive extrusion of endogenous DNA triggers chronic cGAS-STING activation, which inherently acts as a fundamental driver for the epigenetic and metabolic reprogramming of these monocytes, locking them into a hyper-responsive “trained” memory state [33,34]. This STING-dependent trained immunity ensures that upon subsequent cardiovascular stresses, these reprogrammed cells mount an exaggerated, maladaptive pro-inflammatory response. Thus, cGAS-STING signaling does not merely mediate acute local inflammation, but actively orchestrates long-term systemic cardiovascular risk and chronic atherogenesis [31].

Beyond the innate myeloid compartment, the adaptive immune response—particularly the delicate homeostasis between T helper 17 (Th17) cells and regulatory T cells (Tregs)—plays an indispensable role in cardiovascular inflammation [35]. Upon sterile injury, damaged or released endogenous DNA functions as a DAMP that not only activates innate sensors but also indirectly triggers retinoic acid receptor-related orphan receptor gamma t (RORγt)-associated inflammatory pathways [16]. Specifically, DNA-triggered cGAS-STING activation prompts antigen-presenting cells to secrete polarizing cytokines (such as interleukin-1β (IL-1β) and interleukin-6 (IL-6)), which potently induce RORγt expression and drive the differentiation of naïve CD4+ T cells into pro-inflammatory Th17 cells [36,37]. In the cardiovascular milieu, Th17 cells abundantly secrete IL-17A, which exacerbates endothelial dysfunction, accelerates atherogenesis, and promotes pathological fibroblast activation post-myocardial infarction [38,39]. Strictly counterbalancing this inflammatory cascade are Foxp3+ Tregs, which are universally recognized for their indispensable cardioprotective and immunomodulatory functions. Newly recruited and resident Tregs actively secrete potent anti-inflammatory mediators, notably interleukin-10 (IL-10) and transforming growth factor-beta (TGF-β), to directly suppress both hyperactive macrophages and Th17-mediated tissue destruction, limit atheroma progression, and facilitate stable extracellular matrix deposition during myocardial wound healing [40,41]. Consequently, the sustained extrusion of damaged DNA actively disrupts this crucial Th17/Treg balance, driving the chronicity of cardiovascular inflammation and maladaptive heart failure.

Orchestrating these dynamic responses is a complex, anatomically specialized network of resident cardiac immune cells that govern steady-state homeostasis [42]. The healthy myocardium is densely populated by innate immune networks—predominantly dendritic cells and resident macrophages—that strategically reside within the aortic valves, coronary architecture, and the atrioventricular (AV) node [43]. Crucially, these true resident cardiac macrophages are ontogenically distinct from circulating, monocyte-derived populations recruited during acute injury [44]. Deriving primarily from embryonic yolk sac and fetal liver progenitors, these resident macrophages are self-renewing, maintaining their homeostatic populations through local in situ proliferation independent of bone marrow input [42].

This localized immune network extends to the pericardial fluid, which functions as an immunological reservoir harboring innate macrophages, mast cells, and adaptive B cells. During injury, cardioprotective subsets—such as Gata6-expressing macrophages—rapidly mobilize from the pericardial cavity directly into the myocardium to attenuate interstitial fibrosis and preserve ventricular function [45]. Consequently, both resident and newly recruited immune cells exhibit significant temporal plasticity to orchestrate the transition from injury to repair [45,46]. Following myocardial ischemia, early-infiltrating phagocytes initially adopt a highly pro-inflammatory phenotype to clear necrotic debris, subsequently undergoing an essential phenotypic transition toward an anti-inflammatory, pro-resolving state to coordinate stable scar formation [47]. Ultimately, these dynamic immune processes are further shaped by extensive neuro-immune cross-talk [48]. For instance, the localized release of neuropeptides (e.g., substance P and neurotensin) from resident cardiac nerves triggers mast cell degranulation; the ensuing histamine release potently activates fibroblasts, actively driving the structural and fibrotic remodeling of the diseased myocardium [49,50].

## 3. Damaged DNA Extrusion: The Primordial Trigger of Sterile Inflammation in CVD

In the context of cardiovascular disease (CVD), sterile inflammation is fundamentally ignited by the aberrant cytosolic mislocalization of host nucleic acids. Characteristic cardiovascular stressors—including angiotensin II (Ang II)-induced oxidative stress, hyperlipidemia, hyperglycemia, and ischemia—inflict significant structural damage upon both the nuclear and mitochondrial genomes [42,51,52]. Cells initially counter this genotoxic insult via the DNA damage response (DDR), an ATM/ATR-dependent surveillance network that orchestrates diverse repair machineries alongside cell-cycle arrest [53]. However, when these cytoprotective mechanisms are chronically overwhelmed, the ensuing genomic instability establishes a critical intersection with innate immune signaling cascades [54].

Exhaustion of the DDR precipitates the pathological extrusion of endogenous double-stranded DNA (dsDNA) into the cytosol, effectively converting sequestered nucleic acids into potent damage-associated molecular patterns (DAMPs) [55,56]. This mislocalization predominantly proceeds through two distinct subcellular conduits. First, severe stress-induced mitochondrial dysfunction triggers mitochondrial outer membrane permeabilization (MOMP)—often via BAX/BAK oligomerization—allowing inner membrane herniation and subsequent mtDNA efflux; alternatively, oxidative stress can facilitate this release through prolonged mitochondrial permeability transition pore (mPTP) opening or voltage-dependent anion channel (VDAC) oligomerization [57]. Parallel to this, nuclear DNA escapes into the cytoplasm via structural deteriorations. Persistent genotoxic stress generates highly unstable, DNA-containing micronuclei during mitosis that are highly prone to spontaneous rupture [58]. Furthermore, cardiomyocytes subjected to significant mechanical strain (e.g., pressure overload) or senescence can experience direct nuclear envelope ruptures, exposing raw chromatin to the cytosol. The accumulation of this self-DNA is exacerbated by the failure of cytosolic exonucleases; paradoxically, mechanical stress-induced envelope rupture not only overwhelms cytosolic clearance but also facilitates the aberrant nuclear translocation of endoplasmic reticulum (ER)-anchored TREX1, thereby actively perpetuating chronic genomic damage [59].

**Figure 2:**
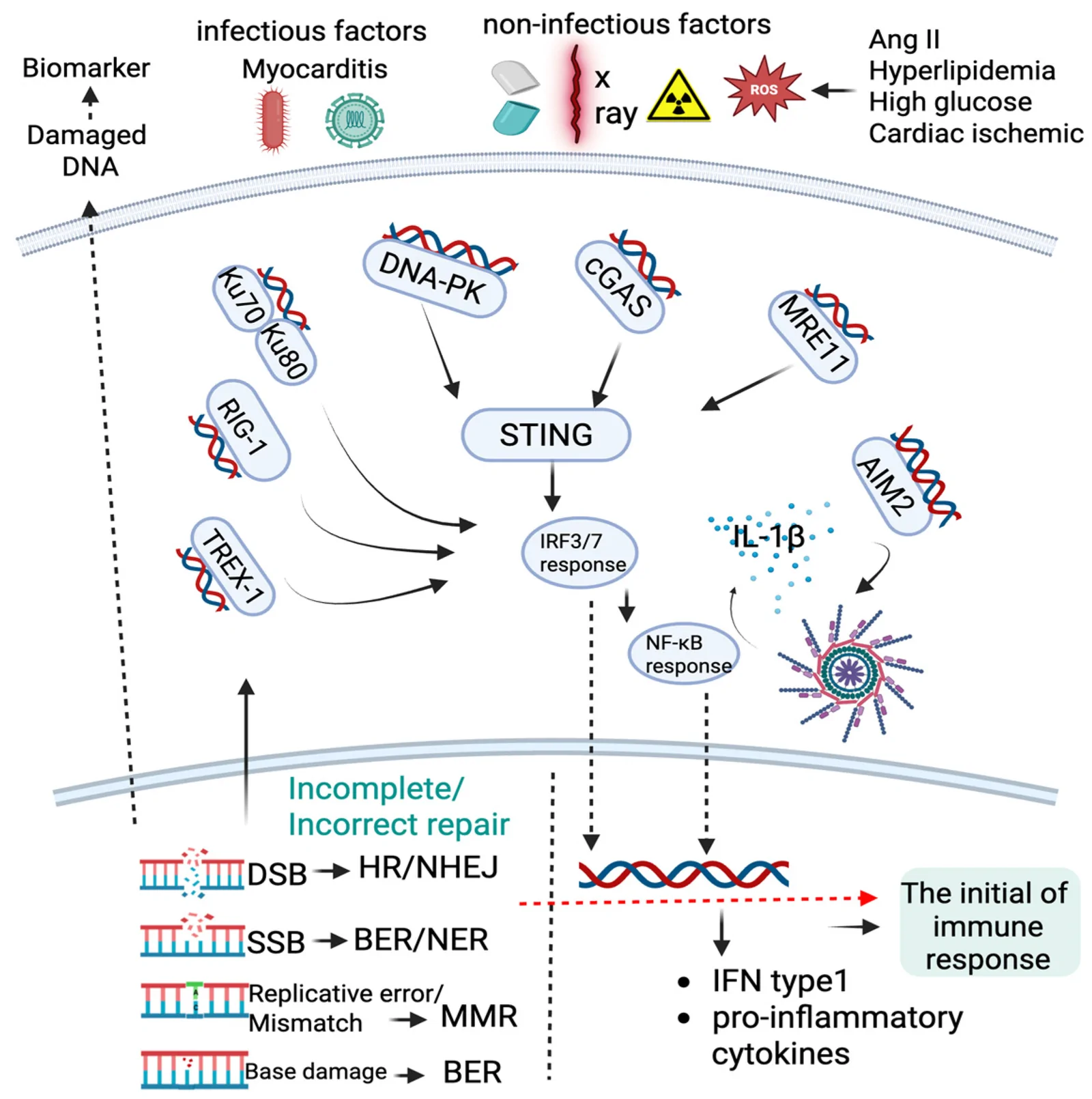
DNA damage and DNA damage response in CVDs. Various stressors induce DNA damage, activating the DDR network (e.g., DNA-PK, MRE11) for repair. Unrepaired DNA leaks into the cytoplasm, engaging sensors (cGAS, AIM2, RIG-I) and effectors (STING) to trigger the release of IFNs and IL-1β. Black arrows indicate the signaling flow from DNA damage to immune activation; red arrows indicate the initiation of immune responses; different colors distinguish stressor types and repair factors. Abbreviations: Ang II, angiotensin II; DDR, DNA damage response; DSB, double-strand break; SSB, single-strand break; HR, homologous recombination; NHEJ, non-homologous end joining; MMR, mismatch repair; BER, base excision repair; cGAS, cyclic GMP-AMP synthase; STING, stimulator of interferon genes; IRF3, interferon regulatory factor 3; NF-κB, nuclear factor kappa B; IL-1β, interleukin-1 beta; ROS, reactive oxygen species; AIM2, absent in melanoma 2; DNA-PK, DNA-dependent protein kinase; Ku70, Ku protein p70 subunit; Ku80, Ku protein p80 subunit; MRE11, meiotic recombination 11; RIG-I, retinoic acid-inducible gene I.

Upon cytosolic accumulation, this misplaced self-DNA is promptly recognized by spatially distinct pattern recognition receptors (PRRs), igniting rapid innate immune activation [16]. While endosomal receptors like TLR9 detect internalized CpG-rich DNA, the cytosolic surveillance network relies heavily on sensors such as absent in melanoma 2 (AIM2) and cGAS to identify aberrant intracellular dsDNA [60]. Moreover, experimental evidence highlights DAI (DLM-1/ZBP1) as an additional cytosolic dsDNA sensor that coordinates the TBK1-IRF3 axis to drive type I interferon expression [61]. Ultimately, the cGAS-stimulator of interferon genes (STING) pathway has emerged as the paramount mechanistic conduit linking cytosolic DNA detection to significant immune activation [62]. Upon binding dsDNA, cGAS synthesizes the second messenger cyclic GMP-AMP (cGAMP), thereby activating STING to orchestrate a robust signaling cascade that unleashes type I interferons (IFNs), pro-inflammatory cytokines, and chemokines—a coordinated cytokine storm that inexorably perpetuates the chronic inflammatory cycle in CVD (Figure 2) [63,64].

## 4. The DNA Sensor cGAS and Activation of the cGAS-STING Pathway

The cyclic GMP-AMP synthase (cGAS) operates as the principal cytosolic sentinel for misplaced double-stranded DNA (dsDNA). Although traditionally characterized as strictly cytosolic, a substantial nuclear cGAS pool is now recognized; however, Severe autoreactivity against the host genome is averted via stringent chromatin tethering that maintains nuclear cGAS in a catalytically inert state [65,66]. Activation of the C-terminal nucleotidyltransferase (NTase) domain is strictly governed by the biophysical properties of the DNA ligand [67]. While cGAS can bind short fragments and RNA-DNA hybrids, robust in vivo activation requires dsDNA exceeding 40 base pairs to facilitate the assembly of higher-order, ladder-like cGAS-DNA networks [68,69]. This length threshold functions as a critical biophysical safeguard, ensuring activation is not spuriously triggered by transient DNA debris, but rather by substantial pathogenic invasion or massive endogenous danger signal extrusion [70]. Upon engaging these activating strands, cGAS undergoes liquid-liquid phase separation (LLPS), generating concentrated, liquid-like micro-compartments [71]. These condensates intrinsically serve a dual purpose: they physically shield the immunostimulatory DNA from TREX1-mediated cytosolic degradation [72], and they optimize the localized oligomerization of cGAS for the highly efficient catalytic conversion of ATP and GTP into the cyclic dinucleotide second messenger, 2′3′-cGAMP [73].

**Figure 3:**
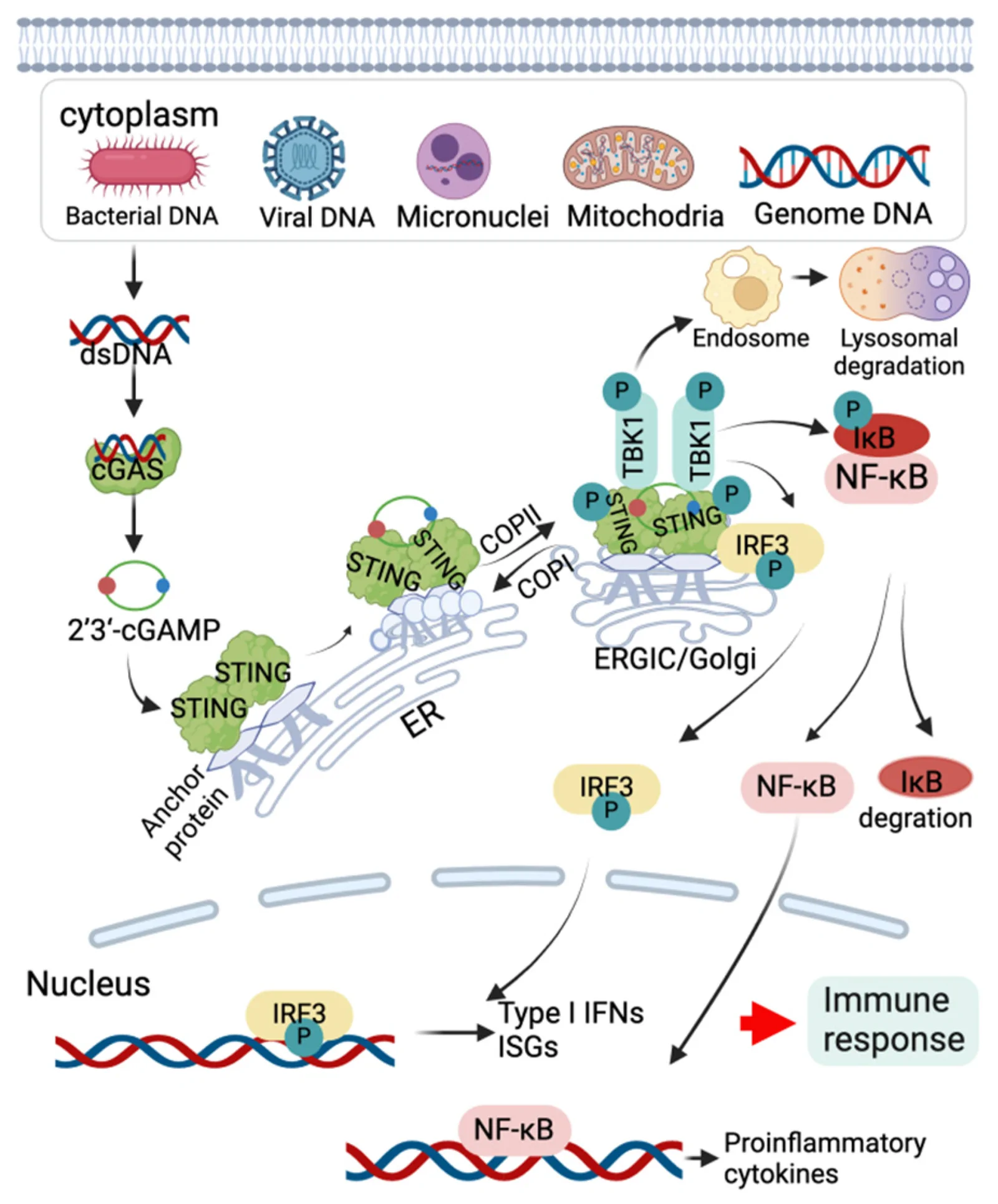
DNA sensor cGAS and classical cGAS-STING pathway. Cytosolic cGAS detects dsDNA to synthesize cGAMP, which activates ER-resident STING. STING translocates to the Golgi, recruiting TBK1 to activate IRF3 and NF-κB. These factors enter the nucleus to drive the expression of IFNs, ISGs, and pro-inflammatory cytokines. Black arrows indicate the direction of signaling transduction; the red arrow indicates the resulting immune response; circles labeled ‘P’ denote phosphorylation. Abbreviations: dsDNA, double-stranded DNA; cGAS, cyclic GMP-AMP synthase; cGAMP, cyclic GMP-AMP; STING, stimulator of interferon genes; ER, endoplasmic reticulum; ERGIC, ER-Golgi intermediate compartment; COPI/II, coat protein complex I/II; TBK1, TANK-binding kinase 1; IRF3, interferon regulatory factor 3; NF-κB, nuclear factor kappa B; IκB, inhibitor of NF-κB; IFNs, interferons; ISGs, interferon-stimulated genes.

This synthesized cGAMP is subsequently detected by the stimulator of interferon genes (STING), an endoplasmic reticulum (ER)-resident transmembrane protein. Binding of cGAMP induces significant conformational rearrangements within the domain-swapped STING homodimer—characterized by a 180° rotation of its ligand-binding domain and the closure of a structural β-sheet “lid”—which catalyze its side-by-side oligomerization [16,74,75]. Activated STING complexes exit the ER and translocate to the Golgi apparatus [76], where they undergo essential palmitoylation at cysteine residues C88 and C91, a requisite modification for downstream effector recruitment [77]. At the Golgi, palmitoylated STING scaffolds TANK-binding kinase 1 (TBK1), culminating in the phosphorylation and subsequent nuclear translocation of interferon regulatory factor 3 (IRF3) to drive the robust transcription of type I interferons and pro-inflammatory cytokines [15,78]. Concurrently, STING potentates the canonical nuclear factor kappa B (NF-κB) network by recruiting the IKK complex, which liberates p65/p50 heterodimers to further amplify the pro-inflammatory gene profile [79,80] (Figure 3).

**Figure 4:**
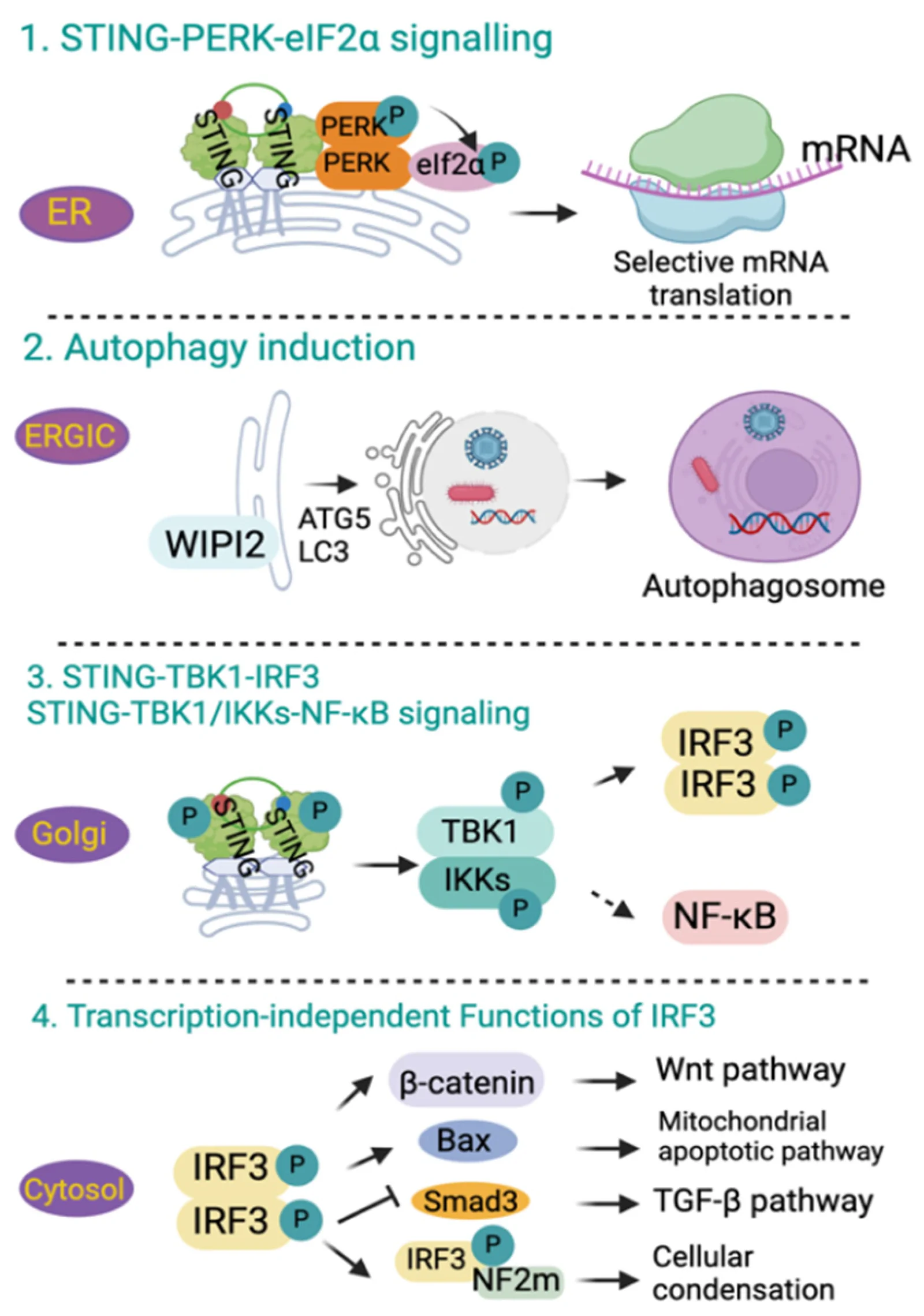
Non-classical cGAS-STING pathway. STING mediates multiple non-canonical pathways: (1) ER-STING activates the PERK-eIF2α translation axis. (2) Translocated STING drives LC3/ATG5-dependent autophagy via WIPI2. (3) Golgi-STING scaffolds TBK1/IKKs to amplify NF-κB. (4) Cytosolic IRF3 modulates apoptosis (via Bax) and TGF-β (via Smad3) signaling independent of transcription. Black solid arrows indicate the direction of signaling transduction or pathway activation; circles labeled ‘P’ denote phosphorylation. Abbreviations: ER, endoplasmic reticulum; ERGIC, ER-Golgi intermediate compartment; PERK, protein kinase R-like ER kinase; eIF2α, eukaryotic initiation factor 2 alpha; ATG5, autophagy related 5; LC3, microtubule-associated protein 1A/1B-light chain 3; WIPI2, WD repeat domain phosphoinositide-interacting protein 2; TBK1, TANK-binding kinase 1; IKK, IκB kinase; IRF3, interferon regulatory factor 3; NF-κB, nuclear factor kappa B; TGF-β, transforming growth factor beta; Bax, Bcl-2-associated X protein.

Beyond these classical cascades, STING orchestrates a distinct regulatory layer via the non-canonical NF-κB pathway. By stabilizing NF-κB-inducing kinase (NIK), STING drives the nuclear accumulation of p52–RelB heterodimers, a process that frequently acts as a crucial negative feedback loop to temper innate sensing and prevent excessive tissue damage [81,82]. The signaling pleiotropy of the cGAS-STING axis extends even further, intersecting with p53, MAPK p38, and STAT3 networks to dictate cellular survival, apoptosis, and intricate cytokine profiles (Figure 4).

The precise regulation of these pathways is of significant clinical significance. While historically investigated within the contexts of oncology [83] and infectious diseases [84], the dysregulation of the cGAS-STING axis is now established as a fundamental engine of sterile inflammation in cardiovascular diseases (CVD) [14]. In obesity-related diabetic cardiomyopathy, for instance, lipotoxic mitochondrial ROS drives cytosolic mtDNA leakage, directly inciting cGAS-STING-mediated myocardial inflammation and apoptosis [85]. Similarly, in models of doxorubicin-induced cardiotoxicity, robust cGAS-STING activation actively exacerbates myocardial apoptosis and cytokine release—a pathology significantly blunted by targeted STING silencing [86]. Notably, an emerging body of evidence delineates critical, STING-independent functions for cGAS in modulating cellular homeostasis, energy metabolism, and tumor dynamics [87,88,89,90]. Within the nucleus, cGAS impedes homologous recombination by interacting with PARP1 to promote tumor growth [87], and drives transcription-independent apoptosis during mitotic arrest, sensitizing cancer cells to anti-mitotic agents [88]. Furthermore, the recognition of cytoplasmic chromatin fragments by cGAS is indispensable for establishing the senescence-associated secretory phenotype (SASP), effectively bridging cellular senescence with chronic tissue inflammation and active tumor surveillance [89,90].

## 5. The cGAS-STING Pathway in Cardiovascular Pathology: From DNA Damage to Immune Dysregulation

**Figure 5:**
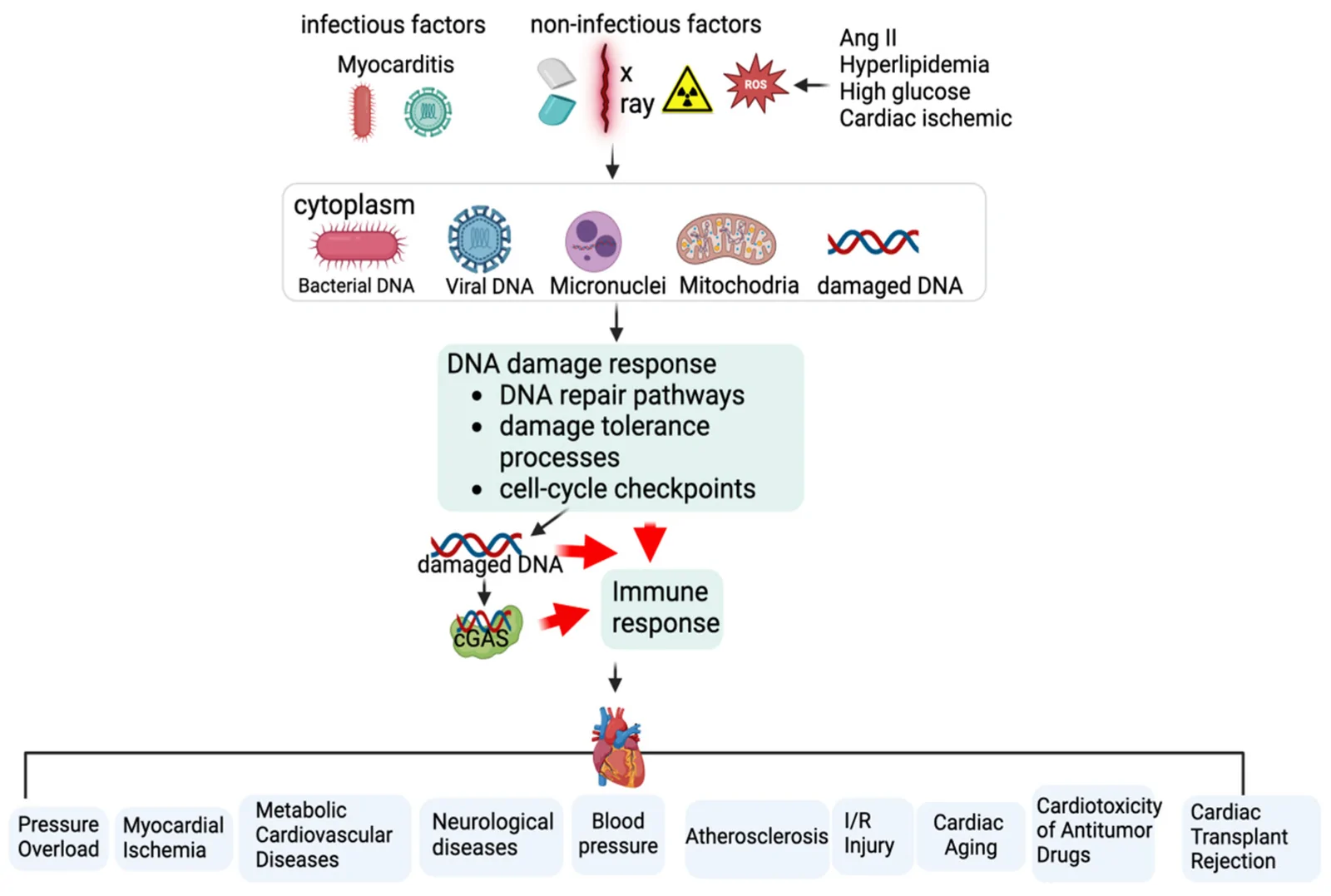
The connection of DNA damage and cGAS-STING pathway in the immune response of CVD. Infectious and non-infectious stressors trigger cytoplasmic DNA accumulation. When DDR fails, this DNA activates cGAS, driving innate immune responses that universally promote diverse cardiovascular pathologies (e.g., atherosclerosis, ischemia, aging). Black arrows denote the sequential progression from DNA damage to cardiovascular pathologies; red arrows denote cGAS-mediated immune activation. Abbreviations: Ang II, angiotensin II; cGAS, cyclic GMP-AMP synthase; CVD, cardiovascular disease; DDR, DNA damage response; I/R, ischemia/reperfusion; ROS, reactive oxygen species; STING, stimulator of interferon genes.

Under pathological cardiovascular conditions, diverse insults—ranging from hemodynamic overload and acute ischemia to lipotoxicity—converge on a singular mechanistic node: the disruption of organelle integrity and the subsequent cytosolic accumulation of nuclear and mitochondrial DNA (mtDNA) [15,91]. This extranuclear DNA serves as the primary activating ligand for the cGAS-STING cascade, a master immunological amplifier that translates localized structural distress into robust sterile inflammation, thereby actively propelling disease pathogenesis (Figure 5).

### 5.1. Hemodynamic and Ischemic Myocardial Injury

#### 5.1.1. Hemodynamic Stress and Pathological Ventricular Remodeling

Sustained hemodynamic overload provokes intense mechanical and neurohormonal stress, culminating in an intracellular reactive oxygen species (ROS) storm. This oxidative burst compromises mitochondrial networks and the nuclear envelope, promoting continuous cytosolic dsDNA efflux. In pressure-overloaded myocardium, inducible nitric oxide synthase (iNOS) critically drives this pathological mtDNA accumulation, igniting a cGAS-STING-dependent inflammatory cascade [92]. This activation stimulates cardiac fibroblast proliferation, extracellular matrix (ECM) deposition, and cardiomyocyte apoptosis, driving maladaptive left ventricular hypertrophy. Endoplasmic reticulum (ER) stress further amplifies this signaling; conversely, targeted cGAS or STING inhibition—via adeno-associated virus (AAV) vectors or gene silencing—significantly blunts macrophage infiltration, preserves contractile function, and improves survival [93,94].

Rather than presenting an unresolved biological paradox, the divergent outcomes of STING signaling in hypertrophy can be synthesized into a framework governed by two primary variables: cell-type specificity and distinct intracellular effector pathways [16,76]. Chronic STING hyperactivation in non-myocytes—specifically resident macrophages and fibroblasts—predominantly drives the maladaptive, NF-κB/IRF3-dependent inflammatory cascades that orchestrate fibrotic remodeling and structural deterioration [92,93]. Conversely, within cardiomyocytes, basal or acute STING signaling interfaces intimately with cellular quality-control machinery. In this specific cellular context, STING overexpression or targeted activation preserves cardiac function by modulating metabolic homeostasis—either by promoting protective mitophagy [95] or by phosphorylating ULK1 to actively restrain excessive, maladaptive autophagy [96]. Thus, whether STING signaling is pathological or protective is largely determined by whether it engages the canonical inflammatory cascade in immune/stromal cells or the autophagic regulatory networks within cardiomyocytes.

#### 5.1.2. Ischemic Heart Disease and Reperfusion Injury

Ischemia/reperfusion (I/R) injury induces catastrophic mitochondrial permeability transition pore (mPTP) opening, resulting in massive mtDNA release into the cytosol, where cGAS operates as a critical damage sensor [97]. In the ischemic microenvironment, mitochondrial dysfunction reproduces a proinflammatory metabolic profile in macrophages and severely blunts their transition to the reparative phase during the post-infarction period [6]. Specifically, cGAS-STING activation traps the local macrophage pool in this pro-inflammatory state, actively preventing essential tissue repair [98]. Consequently, cyclic GMP-AMP synthase loss of function promotes the transformation of macrophages to a reparative phenotype, which results in enhanced repair, significantly improved early survival, and diminished pathological remodeling, including reduced ventricular rupture [99]. Pharmacological STING inhibition (e.g., via H-151) similarly removes this inflammatory blockade, blunts type I interferon responses, and promotes optimal myocardial wound healing [100,101].

To bridge these extensive murine mechanistic findings to clinical pathology, translational efforts have investigated human ischemic heart tissues. However, it is critical to acknowledge that the current human evidence remains purely correlative. Specifically, elevated cGAS-STING pathway markers have been observed in clinical samples; yet, these findings derive from restricted cohorts of explanted failing human hearts obtained during transplantation, serving strictly as correlative indicators of pathway hyperactivation rather than providing direct functional or interventional human data [99]. Furthermore, intercellular communication exacerbates this pathology; small extracellular vesicles (sEVs) secreted by injured cardiomyocytes can transport mtDNA to neighboring cells, activating cGAS-STING in trans to stimulate adverse fibrotic proliferation [102].

### 5.2. Vascular and Metabolic Chronic Stress

#### 5.2.1. Atherosclerosis

In the pro-atherogenic milieu, the vascular endothelium is continuously assaulted by oxidized low-density lipoproteins (ox-LDL), turbulent shear stress, and environmental toxins like cigarette smoke, which collectively trigger severe mitochondrial oxidative stress and DNA fragmentation [64,103]. The sensing of cytosolic dsDNA by endothelial cells and subintimal macrophages upregulates adhesion molecules and accelerates monocyte recruitment, driving plaque progression and vulnerability [104]. Specifically, the accumulation of DNA double-strand breaks activates the cGAS-STING-IRF3 axis, upregulating proinflammatory cytokines and inducing vascular cellular senescence [105]. Scaffold proteins, such as IQGAP1, exacerbate this damage by promoting mtDNA leakage and subsequent cGAS-STING-dependent, NLRP3-mediated endothelial pyroptosis—a process reversible by targeted STING inhibitors like RU.521 or C-176 [106]. Consequently, genetic deletion of STING markedly reduces macrophage infiltration, lipid accumulation, and overall atherosclerotic lesion size [104].

#### 5.2.2. Metabolic Cardiovascular Diseases

Metabolic derangements—including diabetes, obesity, and chronic kidney disease (CKD)—foment chronic low-grade inflammation. Rather than operating through entirely independent pathways, these heterogeneous conditions act as parallel upstream stressors that converge on a singular, unifying hierarchical mechanism: the disruption of mitochondrial network integrity and subsequent mtDNA leakage [107]. In diabetic and obese settings, chronically elevated free fatty acids and hyperglycemia impair mitochondrial bioenergetics, generating excessive ROS. This structural stress is further exacerbated during diabetic ischemia-reperfusion, which compromises mitochondrial dynamics by depleting mitofusin-2 (MFN2), thereby sustaining a pathogenic pool of cytosolic mtDNA [107,108]. Similarly, in CKD, the persistent uremic microenvironment acts as a distinct parallel trigger driving this same sustained mtDNA extrusion [109]. Because cytosolic mtDNA leakage serves as the convergent inflammatory node for these diverse metabolic insults, the endogenous autophagic/mitophagic quality-control machinery emerges as the most therapeutically tractable gatekeeper. When this clearance system is overwhelmed, lipotoxic and uremic mtDNA potently activates the cGAS-STING cascade. This convergent activation subsequently branches into distinct downstream effector networks, triggering myocardial apoptosis, NLRP3 inflammasome-dependent pyroptosis, and, uniquely in the CKD milieu, the transactivation of polyamine metabolism to drive systemic vascular remodeling and cardiac hypertrophy [85,109,110]. Conversely, targeted interventions that restore mitochondrial quality control effectively dismantle this entire signaling hierarchy at its source. For instance, protective secretory proteins like Metrnl ameliorate diabetic cardiomyopathy by enhancing LKB1/AMPK/ULK1-mediated autophagic clearance of damaged mitochondria, effectively inactivating STING prior to signal amplification [111]. Similarly, reversing adverse dietary factors—such as exogenous H2S supplementation to counteract high-choline intake-induced H2S depletion—restores metabolic homeostasis and significantly suppresses the cGAS-STING-NLRP3 axis [112]. Thus, these seemingly distinct single-study mechanisms collectively underscore mitochondrial quality control and mtDNA containment as the unifying therapeutic targets across metabolic cardiovascular diseases.

#### 5.2.3. Cardiac Aging (Senescence)

Cellular senescence involves progressive nuclear lamina degradation, permitting genomic DNA to herniate into the cytosol as cytoplasmic chromatin fragments (CCFs). These CCFs chronically engage the cGAS-STING pathway, establishing it as a fundamental driver of the senescence-associated secretory phenotype (SASP) and organ fibrosis [113]. This process is further exacerbated by an age-related decline in YAP/TAZ mechanotransduction, which unleashes STING-mediated endothelial dysfunction [114]. Clinically, aged human aortic tissues exhibit significantly elevated expression of cGAS, STING, and p-IRF3; crucially, targeted pharmacological or genetic inhibition of this axis successfully attenuates senescence markers and restores endothelial function [115].

### 5.3. Sepsis-Induced Cardiovascular Dysfunction

Severe systemic inflammation induces significant cardiovascular DNA damage via distinct, tissue-specific mechanisms [116]. In the vascular endothelium, cytosolic DNA activates cGAS to synthesize cGAMP, which is exported via multidrug resistance protein 1 (MRP1) and internalized by adjacent vascular smooth muscle cells. This triggers a non-canonical, cGAMP-dependent activation of protein kinase I (PKGI), inducing significant nitric oxide-independent vasorelaxation that acts as a primary driver of life-threatening sepsis-induced hypotension and vascular shock [117,118,119,120]. Concurrently, direct endotoxin (e.g., LPS) assault on the myocardium triggers a massive ROS burst, selectively permeabilizing mitochondrial membranes and spilling mtDNA into the cytosol. This endogenous dsDNA synergizes with circulating pathogenic DNA to hyperactivate cGAS-STING, precipitating severe myocardial apoptosis and contractile dysfunction [119,121]. Interventions that preserve mitochondrial integrity—such as ALDH2 activation via Alda-1—or directly inhibit cGAS (e.g., RU.521) robustly suppress this oxidative and inflammatory cascade, rescuing overall cardiac performance in septic models [122,123].

### 5.4. Iatrogenic and Surgical Triggers

#### 5.4.1. Cardiotoxicity of Antitumor Drugs

Chemotherapeutic agents inflict catastrophic genotoxic stress on the myocardium [124,125]. Doxorubicin directly intercalates into genomic DNA and induces severe mitochondrial oxidative stress [126,127]. This triggers the pathological accumulation of Z-form DNA, which interacts with Z-DNA binding protein 1 (ZBP1) and cGAS to unleash a lethal apoptotic cascade [128]. Furthermore, activation of the cGAS-STING-IRF3 axis in cardiac endothelial cells drives CD38-mediated NAD+ depletion, exacerbating mitochondrial collapse [129]. Similarly, the DNA crosslinking agent cisplatin orchestrates overt cardiac dysfunction via the STING-TNF-α-AP-1 signaling axis [130]. Targeted silencing of STING efficiently blunts these inflammatory and apoptotic cascades, preserving myocardial viability and significantly improving survival rates [86].

#### 5.4.2. Cardiac Transplant Rejection

The ischemia-reperfusion phase intrinsic to heart transplantation, followed by alloimmune recognition, results in significant donor tissue necrosis and massive dsDNA extrusion into the graft microenvironment [131]. This DAMP leakage hyperactivates the graft’s cGAS-STING network, providing an early danger signal that rapidly recruits alloreactive macrophages and CD8+ T cells. Utilizing cGAS-deficient donor tissues or administering pharmacological cGAS inhibitors significantly blunts this alloreactive infiltration and significantly prolongs graft survival, establishing the cGAS-STING axis as a pivotal mechanistic bridge between surgical injury and immune rejection [132,133] (Table 1).

## 6. Crosstalk Between the cGAS-STING Axis and Interconnected Signaling Networks in CVD

The cGAS-STING axis does not operate in pathological isolation; rather, it functions as a central regulatory hub within a multiplexed network that integrates innate immunity, cellular metabolism, and organelle homeostasis. Analyzing cardiovascular inflammation through a strictly linear framework significantly underestimates disease complexity, as the ultimate severity of cardiovascular injury is dictated by the synergistic convergence of these parallel and intersecting signaling cascades [14,134,135].

A primary homeostatic intersection lies between cGAS-STING sensing and the cellular metabolic-autophagic axis. Physiologically, AMP-activated protein kinase (AMPK)—the master metabolic regulator—suppresses sterile inflammation via a dual protective mechanism: it directly phosphorylates and inhibits STING, while concurrently activating ULK1 to drive the p62-dependent autophagic degradation of both cytosolic DNA and STING complexes [136]. However, in ischemic heart disease and metabolic derangements (e.g., diabetic cardiomyopathy or obesity), this AMPK-driven surveillance is significantly suppressed. This metabolic collapse not only derepresses STING but also paralyzes mitochondrial quality control (mitophagy). The ensuing failure to clear dysfunctional mitochondria guarantees continuous mtDNA extrusion, thereby fueling relentless cGAS-STING hyperactivation [14,137,138]. Notably, experimental ablation of critical mitophagy regulators (such as PINK1 or Parkin) precipitates robust, mtDNA-driven systemic inflammation—a pathological phenotype that is completely abrogated by concurrent STING deletion [138].

Beyond metabolic dysregulation, this hyperactivated cGAS-STING network engages in significant, bidirectional crosstalk with the NLRP3 inflammasome to amplify myocardial injury. Activated STING provokes severe endoplasmic reticulum (ER) stress and subsequent lysosomal destabilization, triggering intracellular potassium efflux that acts as an essential secondary catalyst for NLRP3 inflammasome assembly. Concurrently, STING-mediated NF-κB activation—often operating in tandem with parallel endosomal sensors like Toll-like receptor 9 (TLR9)—transcriptionally primes inflammasome components. This synergistic functional coupling inextricably links primary cytosolic DNA sensing to the robust proteolytic maturation and secretion of interleukin-1β (IL-1β) and IL-18, ultimately unleashing widespread myocardial pyroptosis [139].

Ultimately, the cGAS-STING pathway functions as the critical translational node converting metabolic distress and organelle collapse into significant structural cardiovascular damage. Amplified by parallel innate immune sensors and unleashed by failing autophagic clearance, this signaling convergence aggressively propels cardiomyocyte death, fibroblast activation, and maladaptive ventricular remodeling [93,94]. Deciphering these interconnected regulatory nodes is paramount; realizing the therapeutic potential of targeting the cGAS-STING axis in cardiovascular medicine will necessitate sophisticated pharmacological strategies capable of precisely navigating and modulating this intricate immuno-metabolic web.

## 7. The Unique Rationale for Targeting the cGAS-STING Axis over Alternative Pathways

While landmark Phase 3 clinical trials—such as CANTOS and COLCOT—have definitively validated the atheroprotective benefits of anti-inflammatory interventions in humans, these proven clinical strategies predominantly rely on broadly dampening the immune response or inhibiting isolated, downstream cytokines [140,141]. In contrast, we propose a preclinical hypothesis that targeting the cGAS-STING pathway may offer a distinct mechanistic advantage by intervening at the absolute apex of sterile cellular injury. It is crucial to emphasize that no cGAS-STING inhibitor has yet reached comparable clinical-outcome trials in cardiovascular disease; thus, the therapeutic edge of this pathway currently remains an evidence-based preclinical concept rather than a demonstrated clinical superiority. Within these preclinical paradigms, however, neutralizing the inflammatory cascade at its origin—the cytosolic sensing of mislocalized DNA—allows cGAS-STING blockade to simultaneously abrogate divergent downstream pathological networks, encompassing both IRF3-dependent interferon responses and NF-κB-driven cytokine storms. This upstream intervention theoretically circumvents the signaling redundancy and compensatory cytokine production that frequently undermine single-cytokine neutralizing therapies.

Furthermore, the cGAS-STING axis provides exceptional specificity to the precise molecular hallmarks of cardiovascular stress. Unlike broadly engaged pattern recognition receptors (e.g., Toll-like receptors) that respond to a vast array of general danger signals, cGAS is selectively triggered by mitochondrial DNA (mtDNA) extrusion and nuclear chromatin leakage—the definitive structural signatures of acute ischemia, mechanical overload, and lipotoxicity [14,142]. Consequently, tuning this specialized sensor is hypothesized to offer a highly tailored therapeutic window to quell sterile cardiovascular inflammation without broadly compromising systemic antimicrobial immunity.

Crucially, beyond mere immune suppression, modulating the cGAS-STING axis has the unique capacity to actively govern innate immune cell plasticity [98]. While historically conceptualized as a binary shift from a pro-inflammatory state to a tissue-reparative phenotype [98], contemporary single-cell transcriptomic literature increasingly characterizes macrophage polarization as a fluid, multidimensional continuum [143]. In this more nuanced context, rather than acting as a simple binary switch, targeted inhibition of the cGAS-STING pathway shifts the macrophage population spectrum away from deleterious inflammatory states and actively promotes phenotypic signatures associated with tissue repair, thereby orchestrating robust myocardial wound healing [15,143]. Consistent with this continuous phenotypic modulation, single-cell transcriptomic profiling in murine infarction models demonstrates that interrupting the cGAS-STING-IRF3 cascade specifically abolishes the type I interferon response within cardiac macrophages. This targeted cellular blockade reduces inflammatory infiltration, attenuates pathological ventricular dilation, and improves overall post-infarction survival [15].

## 8. The Translational Horizon of cGAS-STING Pathway Inhibitors

Although STING agonists have revolutionized immuno-oncology by actively stimulating anti-tumor immunity [144], the therapeutic imperative in cardiovascular disease (CVD) necessitates a fundamentally divergent approach. Translating the cGAS-STING axis into cardiovascular therapeutics fundamentally relies on the development of potent, highly selective antagonists capable of quenching chronic sterile inflammation without globally compromising essential innate host defenses.

Substantial progress has been achieved in targeting the apex of this cascade through direct cGAS inhibition. Small-molecule catalytic site occupants—such as PF-06928215, RU.521, and X6—effectively paralyze cGAS enzymatic activity [145,146,147] (Table 2). Alternatively, competitive antagonism can be achieved using A151, a suppressive oligodeoxynucleotide containing repetitive TTAGGG motifs that physically obstructs cytosolic dsDNA from engaging the cGAS enzyme [148]. Crucially, this structural blockade is supported by human-specific validation, robustly abrogating downstream type I interferon production in human monocytes [148] and mitigating neuroinflammation in experimental ischemic stroke [149]. Beyond novel synthetics, drug repositioning offers established clinical agents like quinacrine and aspirin, which exhibit off-target cGAS-inhibitory properties [150,151]. Furthermore, natural compounds like epigallocatechin gallate (EGCG) blunt DNA-triggered autoinflammation by disrupting the cGAS-G3BP1 interaction [152].

Parallel pharmacological innovations have successfully targeted STING, the downstream signaling hub. Nitrofuran derivatives—notably C-176, C-178, and their optimized analog H-151—have emerged as highly potent antagonists capable of suppressing cytokine production. Mechanistically, these compounds covalently modify the Cys-91 residue of STING, abolishing Golgi-dependent palmitoylation indispensable for STING multimerization [77,153]. Mechanistically, although physiological STING activation relies on the combined palmitoylation of both Cys-88 and Cys-91 [77], biochemical profiling establishes that these specific antagonists exhibit strict single-residue specificity. They covalently modify exclusively the Cys-91 residue, with no concurrent interaction at Cys-88 [153]. This highly targeted, single-site blockade at Cys-91 is mechanistically sufficient to sterically abrogate the entire Golgi-dependent palmitoylation process indispensable for STING multimerization [77,153]. However, the translational viability of these agents varies significantly due to interspecies structural divergence. C-176 and C-178 selectively bind murine STING but fail to suppress the human ortholog; in contrast, H-151 was explicitly developed to overcome this barrier, demonstrating robust cross-species efficacy in both murine models and human cells [153]. In the context of ischemic injury, targeted administration of H-151 actively suppresses TBK1 phosphorylation within infiltrating macrophages, preventing secondary cardiomyocyte apoptosis and attenuating myocardial fibrosis [101]. To overcome pharmacokinetic limitations, sophisticated nanocarrier systems have been engineered, such as loading C-176 into polyethyleneimine-coated mesoporous polydopamine nanoparticles (PEI-PDA@C-176 NPs) to selectively target local macrophages [154]. Because this specific nanoplatform currently utilizes the murine-specific C-176, its efficacy remains strictly preclinical, necessitating the substitution with human-validated antagonists for future translational development.

Beyond direct structural antagonism, manipulating the intricate immuno-metabolic interface presents a Substantial alternative. Nitro-fatty acids (NO2-FA) operate as pleiotropic STING suppressors by modifying key regulatory nodes—such as unleashing the Keap1-Nrf2 antioxidant axis—while simultaneously dismantling NF-κB-driven signaling [155]. Crucially, NO2-FA successfully crosses the interspecies barrier, effectively dampening type I interferon production even in primary human fibroblasts derived from patients with SAVI (STING-associated vasculopathy with onset in infancy) [155]. This multifaceted modulatory landscape is further expanded by the cyclopeptide Astin C, which exhibits validated efficacy in blocking STING-IRF3 cascades in human macrophage cell lines [156,157]. Conversely, early immuno-metabolic modulators like C-18 [158] have been shown to lack sufficient binding affinity or inhibitory activity against human STING, underscoring the high translational attrition rate in this domain [159]. Ultimately, achieving optimal clinical translation in CVD will require human-optimized modulators alongside broader physiological interventions to comprehensively dismantle the chronic autoinflammatory loop [160,161,162].

**Table 2:** The Pharmacological Landscape and Translational Horizon of cGAS-STING Inhibitors.

Agent/Compound	Target Node	Mechanism of Action	Preclinical Applications & CVD Specificity	Translational Limitations & Species Validation
PF-06928215, RU.521, X6 [145,146,147]	cGAS catalytic site	Competitive catalytic inhibition.	Sepsis (rescues cardiac function). CVD-related systemic model	Poor solubility/clearance; RU.521 is human/murine cross-species validated.
A151 [14,16]	dsDNA/cGAS interface	Physical obstruction of dsDNA binding.	Ischemic stroke (mitigates infarct expansion). Extrapolated from neurovascular model	Poor pharmacokinetics; requires localized delivery. Validated in human monocytes.
EGCG, Quinacrine, Aspirin [150,151,152]	cGAS (Allosteric/Off-target)	Off-target inhibition or allosteric disruption (EGCG).	DNA-triggered autoinflammation. Extrapolated from non-CVD autoimmune models.	Kinetic challenges; risks impairing normal wound healing.
C-176, C-178, H-151 [106,153]	STING Cys-91 residue	Blocks palmitoylation and multimerization.	Ischemic injury (reduces fibrosis/IFN signaling). Direct CVD-specific validation	C-176/C-178 lack human potency (~68% identity barrier). H-151 is human-validated.
PEI-PDA@C-176 NPs [154]	Extracellular DNA & STING	Scavenges extracellular DNA & delivers STING inhibitors.	Targeted delivery overcomes pharmacokinetic limits. Extrapolated from general inflammatory models	Requires precision to bypass hepatic toxicity/infections. Relies on C-176 (murine-validated only).
NO2-FA, Astin C, RTA-408, Itaconate [155,156,163,164]	Immuno-metabolic interface	Pleiotropic suppression (Nrf2/PPARγ activation, NF-κB blockade).	Broad inflammatory suppression. Extrapolated from non-CVD models	Systemic immunocompromise (infection/malignancy risk). Both demonstrate human-specific validation.
C-18, RTA-408, Itaconate [158,159,163,164]	Immuno-metabolic interface	Pleiotropic suppression.	Broad inflammatory suppression. Extrapolated from non-CVD models	C-18 lacks human STING activity. Others require further human-specific evaluation.

## 9. Challenges and Limitations in Clinical Translation

Despite the Substantial preclinical rationale for cGAS-STING modulation, the trajectory toward routine cardiovascular clinical practice is obstructed by Significant translational barriers. Several pathway modulators are currently undergoing early-phase evaluation in oncology and autoimmune contexts, cardiovascular applications remain firmly embedded in the preclinical and early translational stages. Chief among these are the suboptimal pharmacokinetic and pharmacodynamic profiles of first-generation modulators [16]. Characterized by poor aqueous solubility, restricted oral bioavailability, and rapid systemic clearance, these early agents frequently necessitate localized or intratumoral administration—a delivery paradigm that is fundamentally incompatible with the systemic and widely disseminated nature of cardiovascular pathologies [153].

Beyond these physicochemical limitations, translational efforts are significantly confounded by significant interspecies structural divergence. Human and murine STING proteins share merely ~68% sequence identity, dictating significant discrepancies in ligand-binding affinities and pocket architectures. For instance, molecular dynamics reveal that a single amino acid variation in the human STING lid region maintains an open-inactive conformation, inherently precluding the binding of canonical murine-active agonists such as DMXAA [165]. Consequently, compounds demonstrating robust efficacy in murine models frequently exhibit precipitous drops in potency, altered binding kinetics, or unpredictable off-target effects in human cohorts, driving high attrition rates in early-phase development [153]. Circumventing this Significant species barrier mandates rigorous cross-species validation; notably, the small molecule RU.521 has emerged as an Representative, translatable scaffold, demonstrating equipotent suppression of cGAMP production across both human and murine primary cells [166]. However, it is critical to clarify that this cross-species validation has predominantly been established in general primary immune cells, such as macrophages. To date, rigorous testing of RU.521 and similar scaffolds within cardiovascular-specific human models—such as primary human cardiomyocytes or engineered human heart tissues—remains largely unexplored. Consequently, extrapolating its generalized immunological efficacy to specific cardiovascular translational viability still requires definitive, tissue-specific validation.

Complicating this pharmacological landscape is the spatiotemporal pleiotropy of the cGAS-STING axis, which renders the definition of an optimal therapeutic window exceptionally challenging. While chronic signaling inexorably drives maladaptive cardiac remodeling and fibrogenesis, basal pathway activation is indispensable for the immediate post-ischemic clearance of necrotic debris and the orchestration of primordial tissue repair [160]. Thus, mapping the precise spatiotemporal kinetics required to quench chronic sterile inflammation without inadvertently paralyzing essential physiological wound healing remains a critical unmet clinical need [14].

Equally paramount is the inherent danger of systemic immunocompromise. The global pharmacological blockade of this primordial innate immune sentinel threatens to render hemodynamically frail cardiovascular patients highly vulnerable to opportunistic viral and bacterial pathogens, or potentially unmask latent malignancies [16,84]. This critical safety concern is strongly underscored by analogous experience with biologic anti-inflammatory therapies already evaluated in cardiovascular populations. For instance, in the landmark CANTOS trial, while targeted interleukin-1β inhibition with canakinumab successfully reduced cardiovascular events, it was simultaneously associated with a statistically significant increase in fatal infections and sepsis [140]. To mitigate these profound risks, future clinical trial designs evaluating systemic cGAS-STING antagonists must incorporate rigorous infection and malignancy surveillance strategies. Such protocols should mandate comprehensive baseline screening for latent infections (e.g., tuberculosis and chronic viral hepatitis) and occult malignancies, coupled with high-frequency longitudinal monitoring of leukocyte differentials and acute-phase reactants to rapidly detect emerging immunosuppression. Overcoming these multifaceted translational hurdles will ultimately necessitate the synergistic integration of these strict clinical surveillance frameworks with next-generation, human-optimized modulators and precision nanomedicine (e.g., macrophage-targeted or myocardium-homing delivery platforms), alongside the discovery of robust prognostic biomarkers to guide rigorous patient stratification [14].

An additional, yet profoundly significant, translational limitation is the widespread underrepresentation of sex as a biological variable (SABV) in current cGAS-STING research. The vast majority of the foundational preclinical studies cited herein—particularly the murine models evaluating myocardial infarction and hemodynamic overload—predominantly or exclusively utilized male cohorts to mitigate hormonal variability [6,99]. This introduces a critical translational blind spot, as robust clinical and epidemiological evidence [167,168] establishes that innate immune signaling exhibits profound sex-based dimorphism in cardiovascular pathology. Mechanistically, females generally mount a significantly more aggressive type I interferon response and exhibit divergent macrophage polarization dynamics compared to males. This dimorphism is driven in large part by the immunomodulatory effects of estrogen receptor signaling and incomplete X-chromosome inactivation, which influences the dosage of several downstream immune-regulatory genes. Consequently, the activation threshold of the cGAS-STING cascade, the magnitude of the resulting cytokine storm, and the ultimate therapeutic efficacy of STING antagonists are highly likely to be sex-dependent. Addressing this gap by rigorously incorporating both sexes in preclinical mechanistic models and stratifying future human clinical trials by sex remains an indispensable prerequisite for the safe and effective translation of personalized cGAS-STING immunotherapies.

## 10. Future Perspectives: Precision Medicine Targeting the DNA Damage-Immune Axis

Moving beyond the monolithic pharmacological blockade of the cGAS-STING axis, realizing the clinical potential of this network necessitates a multifaceted precision medicine paradigm. To align the depth of our mechanistic understanding with actionable translational strategies, future investigative efforts must prioritize three fundamental pillars:

### 10.1. Pharmaco-Engineering: Biomimetic Delivery and Next-Generation Scaffolds

To circumvent the poor pharmacokinetics and systemic toxicity of first-generation modulators, future pharmacological pipelines must pivot toward human-optimized scaffolds and precision nanomedicine. While advanced targeted molecules like H-151 successfully overcome the interspecies structural barrier [153], controlling their spatiotemporal deployment remains paramount. Engineered extracellular vesicles (EVs) and naturally derived outer membrane vesicles (OMVs) represent a highly promising next-generation compound delivery class [83,169]. By exploiting these biomimetic conduits, STING inhibitors can be strictly steered toward inflamed myocardial beds or specific resident macrophage subpopulations [170]. This spatiotemporally regulated targeting effectively bypasses off-target hepatic toxicity and preserves systemic antimicrobial defenses [14,171]. Furthermore, integrating these targeted platforms with upstream genotoxic mitigators (e.g., angiotensin receptor blockers (ARBs) and angiotensin-converting enzyme inhibitors (ACEIs)) offers a synergistic compound strategy to simultaneously preempt DNA fragmentation and quench downstream STING hyperactivation [172,173].

### 10.2. Precision Stratification: Epigenetic and Transcriptomic Biomarker Signatures

Translating these immunomodulators into human cohorts requires robust prognostic biomarkers to identify the specific cardiovascular subpopulations most receptive to therapy. First, circulating cell-free mitochondrial DNA (cf-mtDNA) and plasma cGAMP quantification offer highly specific, real-time readouts of upstream organelle damage and active cGAS engagement [14]. Second, transcriptomic interferon-stimulated gene (ISG) signatures derived from peripheral blood mononuclear cells (PBMCs) can reliably stratify patients locked in a STING-driven hyperinflammatory endotype. Complementing this, deciphering patient heterogeneity through epigenetic profiling—primarily DNA methylation and histone acetylation marks governing innate immune networks—provides a dual translational advantage: mapping these signatures yields predictive biomarkers, while therapeutically targeting the governing epigenetic enzymes offers a novel mechanistic avenue to recalibrate cGAS-STING hyperreactivity [174,175].

### 10.3. Trial Design Architecture: Spatiotemporal Windows and SABV Integration

Transitioning these biological insights into human cardiovascular trials demands meticulous protocol design to ensure both efficacy and safety. First, addressing the spatiotemporal pleiotropy of cGAS-STING is critical; trial windows must be carefully calibrated to avoid the acute post-ischemic phase—where basal STING activation is indispensable for necrotic clearance—and focus specifically on the chronic, maladaptive remodeling phase [160]. Second, informed by the safety outcomes of the CANTOS trial, protocols must mandate comprehensive baseline screening for latent infections and occult malignancies, coupled with high-frequency longitudinal leukocyte monitoring to mitigate severe immunosuppression risks [140]. Finally, future trial designs must rigorously incorporate sex as a biological variable (SABV) in their randomization and primary endpoint analyses, given the established estrogen- and X-chromosome-driven dimorphism in type I interferon responses that dictates STING activation thresholds [168].

By transitioning from broad-spectrum immunosuppression toward these precision-engineered, biomarker-guided, and spatiotemporally calibrated interventions, the cardiovascular field can definitively harness the therapeutic promise of the cGAS-STING axis while securing uncompromising patient safety.

## 11. Conclusions and Future Perspectives

The pathological accumulation of cytosolic DNA has emerged as a fundamental danger signal, inextricably linking subcellular organelle damage to macroscopic sterile inflammation across the cardiovascular disease (CVD) spectrum. Operating at the nexus of this response, the cGAS-STING axis functions as a master immunological rheostat. Although basal activation of this primordial innate sensor is evolutionarily conserved to orchestrate essential tissue repair and host defense, its chronic, unchecked hyperactivation inexorably drives maladaptive ventricular remodeling, severe ischemic injury, and progressive atherogenesis. Furthermore, the significant integration of cGAS-STING signaling with core cellular machineries—particularly the autophagic and metabolic nutrient-sensing networks—cements its status as a central, pleiotropic hub in cardiovascular pathophysiology.

Despite the Substantial therapeutic efficacy of cGAS-STING modulation in preclinical paradigms, the leap toward routine clinical application remains constrained by Significant pharmacological and physiological barriers. To bridge this translational chasm, future investigative efforts must pivot toward precision spatiotemporal targeting. The engineering of advanced delivery architectures—encompassing ischemia-responsive prodrugs, macrophage-targeted nanomedicines, and localized bio-devices—is paramount to decisively quench cardiac autoinflammation without broadly paralyzing systemic immunocompetence. Equally critical is the rigorous delineation of optimal therapeutic windows; clinical strategies must meticulously balance the physiological imperative of acute, STING-mediated necrotic clearance (e.g., immediate post-infarction) against the significant hazards of its sustained hyperactivation. Ultimately, synergizing these targeted interventions with robust prognostic biomarkers—ranging from circulating cell-free DNA dynamics and plasma cGAMP quantification to transcriptomic interferon-stimulated gene (ISG) signatures—will enable rigorous, data-driven patient stratification. Only through this cohesive, precision-guided framework can the immense therapeutic promise of modulating the DNA damage-immune axis be fully realized in contemporary cardiovascular medicine.

## Figures and Tables

**Table 1:** Synthesizing Evidence Maturity of cGAS-STING Mechanisms in Cardiovascular Contexts.

Disease Context	Key Mechanistic Claim	Evidence Maturity	Representative Citations
Ischemia & Myocardial Infarction	Arrests macrophages in pro-inflammatory state; blunts tissue repair	High/Replicated: Validated across multiple independent groups, pharmacological/genetic models, and human samples.	[97,98,99,100,101]
Hemodynamic Overload	Drives iNOS-dependent fibroblast proliferation and apoptosis.	High/Replicated: Supported by AAV vectors, gene silencing, and various myopathic models.	[92,93,94,95,96]
Atherosclerosis	Induces endothelial senescence and plaque vulnerability via IRF3/NLRP3.	High/Replicated: Demonstrated via STING inhibitors (RU.521/C-176) and genetic deletion by distinct groups.	[16,64,103,104,105,106]
Doxorubicin Cardiotoxicity	Z-form DNA triggers Severe apoptosis and NAD+ depletion.	High/Replicated: Verified by multiple labs using in vitro and in vivo DOX models.	[124,125,126,127,128,129]
Sepsis-Induced Dysfunction	Promotes vasorelaxation via cGAMP export; induces myocardial apoptosis.	Moderate/Replicated: Confirmed across distinct vascular and myocardial readouts.	[117,118,119,120,121,122,123]
Diabetic Cardiomyopathy	Glucolipotoxicity triggers STING-dependent pyroptosis.	Moderate: Broad metabolic findings, though specific pathways vary (e.g., MFN2 vs. Metrnl).	[85,107,108,110,111]
CKD-Associated Remodeling	Uremic milieu transactivates polyamine metabolism.	Emerging/Single Study: Currently reliant on a specific isolated preclinical study.	[109]
High-Choline Diet Injury	H2S depletion triggers cGAS-STING-NLRP3 cascade.	Emerging/Single Study: Currently reliant on a specific isolated preclinical study.	[112]

## Data Availability

No new data were created or analyzed in this study. Data sharing is not applicable to this article.
